# Seasonal dynamics of trace elements, sediment contamination, and child health risks in six Egyptian red sea ports: an integrated water–sediment–human health assessment

**DOI:** 10.1007/s10653-026-03398-z

**Published:** 2026-08-07

**Authors:** Ibrahem M. Abdallah, Lamiaa I. Mohamedein, Khalid M. El-Moselhy, Mohamed A. El-Sawy

**Affiliations:** National Institute of Oceanography and Fisheries (NIOF), Cairo, Egypt

**Keywords:** Arsenic, Trace elements, Human health risk assessment, Ecological risk assessment, Coastal sediments, Red Sea, Egyptian ports, Source apportionment

## Abstract

Coastal port environments along the Red Sea–Suez Canal corridor face intensifying multi-stressor pressures, yet integrated seawater–sediment–human-health assessments of toxicologically distinct trace elements remain scarce. In this study, arsenic (As), selenium (Se), tin (Sn) and mercury (Hg) were quantified in surface seawater (*n* = 38) and surficial sediments (*n* = 19) across six Egyptian ports (Port Tawfiq, Zayteiat, Hurghada, Safaga, Nuweibaa and Sharm El-Sheikh). Six contamination and ecological-risk indices (CF, Igeo, PLI, MPI, Er and RI) were integrated with a USEPA-compliant human health risk assessment and multivariate source apportionment to disentangle natural and anthropogenic signatures. Dissolved As exhibited a pronounced and biogeochemically coherent seasonal inversion, with winter concentrations (8.82 ± 1.04 µg L^−1^) 3.2-fold higher than summer values (2.76 ± 0.53 µg L^−1^; *p* < 0.001), whereas Se followed the opposite trend (summer > winter; *p* < 0.001). All stations fell within the “low” ecological-risk category, with sediment-based indices uniformly low (PLI = 0.07–0.47; RI = 3.6–22.4). Critically, non-carcinogenic hazard indices for children exceeded unity at all six ports (HI = 1.13–1.57), driven predominantly (> 92%) by As, an exceedance that would be masked by adult-only assessment (HI = 0.50–0.67). Total carcinogenic risk remained within the acceptable 10^−6^–10^−4^ range. Principal component analysis and inter-element correlations (*r* ≥ 0.84, *p* < 0.001) resolved two anthropogenic clusters: a legacy antifouling-paint (Sn–As–Hg) signature at high-traffic ports persisting over a decade after the 2008 IMO organotin ban, and petroleum-related Se enrichment at the Zayteiat oil terminal. These findings highlight the necessity of age-stratified exposure assessment and sustained multi-compartment monitoring as Suez Canal traffic intensifies.

**Supplementary Information:**

The online version contains supplementary material available at 10.1007/s10653-026-03398-z.

## Highlights

- Winter dissolved As reached 12.4 µg L^−1^ at Port Tawfiq, 3.2 t higher than summer values (*p*  < 0.001).
- Hakanson RI values were < 30 at all 19 stations, indicating low ecological risk; CF and PLI remained below unity except for As at Port Tawfiq.
- Children's hazard index exceeded 1 at all six ports (HI = 1.13–1.57), driven primarily by arsenic exposure.
- Strong sediment As–Sn–Hg correlations (*r* ≥ 0.84, *p* < 0.001) suggest a legacy antifouling-paint signature.
- Integrated seasonal and multi-index analyses indicate persistent antifouling-related tin contamination more than a decade after the 2008 IMO organotin ban.

**Supplementary Information:**

The online version contains supplementary material available at 10.1007/s10653-026-03398-z.

## Introduction

Port environments constitute among the most spatially concentrated point-source receivers of anthropogenic trace elements in the coastal ocean, integrating cargo handling, shipyard maintenance, antifouling biocide leachate, oil-terminal discharges, ballast water, dredge-spoil resuspension and urban runoff over comparatively small geographic footprints (El-Sorogy et al., 2020; Lagerström et al., 2020; Sayed et al., 2025). Because trace elements are non-biodegradable, persistent in sediments and capable of biomagnification through coastal food webs, port-specific assessments are increasingly framed as an interconnected water–sediment–biota–human-health continuum rather than single-compartment surveys (Elgendy et al., 2024; El-Sawy et al., 2023; Maeyouf et al., 2025). The 2008 entry-into-force of the IMO International Convention on the Control of Harmful Anti-fouling Systems on Ships (AFS Convention) prohibited the use of organotin biocides on vessel hulls (IMO International Maritime Organization. 2008. International Convention on the Control of Harmful Anti-Fouling Systems on Ships (AFS Convention). Adopted 5, 2008), yet recent investigations from the Baltic Sea and South Atlantic continue to detect butyltin signals in port sediments and water columns more than a decade after the ban (Bełdowska et al., 2023; Nudi et al., 2024), demonstrating both the longevity of legacy contamination and the analytical sensitivity now required to evaluate compliance.

The Egyptian Red Sea coast, bracketed by the Suez Canal at its northern terminus and the Bab-el-Mandeb strait to the south, represents a geo-strategically critical maritime corridor through which approximately 12–15% of global seaborne trade transited prior to the 2024 Red Sea shipping disruption (UNCTAD United Nations Conference on Trade & Development, 2024). Six commercial and tourism ports (Port-Tawfiq, Zayteiat, Hurghada, Safaga, Nuweibaa and Sharm El-Sheikh) collectively handle petroleum, phosphate, container, passenger and cruise traffic, and discharge into a semi-enclosed, oligotrophic basin whose limited freshwater input and high evaporation regime favour accumulation rather than dilution of dissolved contaminants (Mohamedein et al., 2023; Nour et al., 2022). Recent regional assessments have documented heavy-metal enrichment in sediments of the Gulf of Aqaba (El-Sorogy et al., 2020), the Gulf of Suez coastal zone (El-Sawy et al., 2023; El-Sorogy et al., 2024) and Hurghada nearshore sediments (El-Sorogy et al., 2024), but these have focused predominantly on the conventional metal suite (Cu, Pb, Zn, Cd, Ni, Cr, Fe, Mn) and have rarely treated arsenic, selenium, tin and mercury as a coherent toxicological cluster.

Each of these four elements occupies a distinctive niche in marine pollution science. Arsenic is a Group 1 human carcinogen whose dissolved concentrations in seawater are mediated by phytoplankton uptake via the phosphate analogue pathway, microbial methylation to dimethylarsenate (DMA) and arsenosugars, and redox-sensitive partitioning between AsV and AsIII (Khan et al., 2023; Wang et al., 2021; Yu et al., 2025); seasonal cycling in temperate and subtropical seas typically produces summer minima driven by bloom uptake and winter maxima reflecting remineralization. Selenium is an essential micronutrient at sub-µg L^−1^ concentrations but rapidly transitions to toxicity at moderately elevated levels, with a narrow window between deficiency and harm; it co-occurs with hydrocarbons in crude petroleum and is therefore a useful chemical tracer of oil-terminal influence (Breuninger et al., 2023; Mihaljevič et al., 2024). Tin in port environments may originate from legacy antifouling coatings, including TBT residues and degradation products, as well as other anthropogenic sources; despite the AFS ban, TBT persists in port sediments with reported half-lives of 9 days (oxic surface) to > 100 days (anoxic, cold-temperate winter conditions) (Agency for Toxic Substances & Disease Registry)., 2005; Bełdowska et al., 2023). Mercury cycling is sensitive to sea-surface temperature and biogeochemical conditions, which may influence dissolved-phase concentrations and methylmercury formation (Wang et al., 2023; Zhou & Zhang, 2024).

The foundational seawater–sediment dataset for these four elements across the six Egyptian Red Sea ports was established by Mohamedein et al. (2023), who reported metal pollution index (MPI) and geoaccumulation index (I_geo_) values. That study, however, did not deploy a Hakanson-based potential ecological risk framework, did not perform a USEPA-compliant human-health risk assessment, and did not apply multivariate source apportionment analytical approaches that are increasingly employed in contemporary pollution assessment studies (Khaled-Khodja et al., 2024; Oza et al., 2025). Three questions therefore remain unresolved for this regionally and economically important system. The magnitude of non-carcinogenic and carcinogenic risk to port-area populations (particularly children) via direct seawater ingestion at recreational beaches has not been quantified, despite the fact that exposure parameters scaled to body weight often produce higher paediatric hazard quotients than adult equivalents because of lower body weight and higher exposure rates. It also remains unclear whether the joint spatial and seasonal pattern of As, Se, Sn and Hg can distinguish antifouling-paint inputs from petroleum operations using principal component analysis (PCA) and inter-element correlation structure. Finally, few studies have systematically compared these Egyptian Red Sea ports with international benchmarks for port-sediment contamination in the post-AFS-ban era.

Building on the Mohamedein et al. (2023) baseline, this study addresses these gaps by: (i) re-examining the spatial–temporal distribution of As, Se, Sn and Hg across 19 stations with an expanded index suite including contamination factor (CF), pollution load index (PLI), Hakanson ecological risk factor (E_r_) and potential ecological risk index (RI); (ii) deriving age-stratified, USEPA-compliant non-carcinogenic and carcinogenic risk estimates for adults and children via the oral seawater ingestion pathway; (iii) applying PCA and Pearson correlation analysis to identify source clusters; and (iv) benchmarking the results against contemporaneous Red Sea, Mediterranean, Persian Gulf and global port datasets to place the Egyptian Red Sea system in regional and international context. The work is timely: as Suez Canal traffic recovers from the 2024 disruption (UNCTAD United Nations Conference on Trade & Development, 2024) and ship-call density returns toward pre-disruption levels, port-area trace-element loads are likely to rebound, making a robust, methodologically integrated baseline both scientifically defensible and operationally useful for environmental management.

## Materials and methods

### Study area and sampling design

**Fig. 1:**
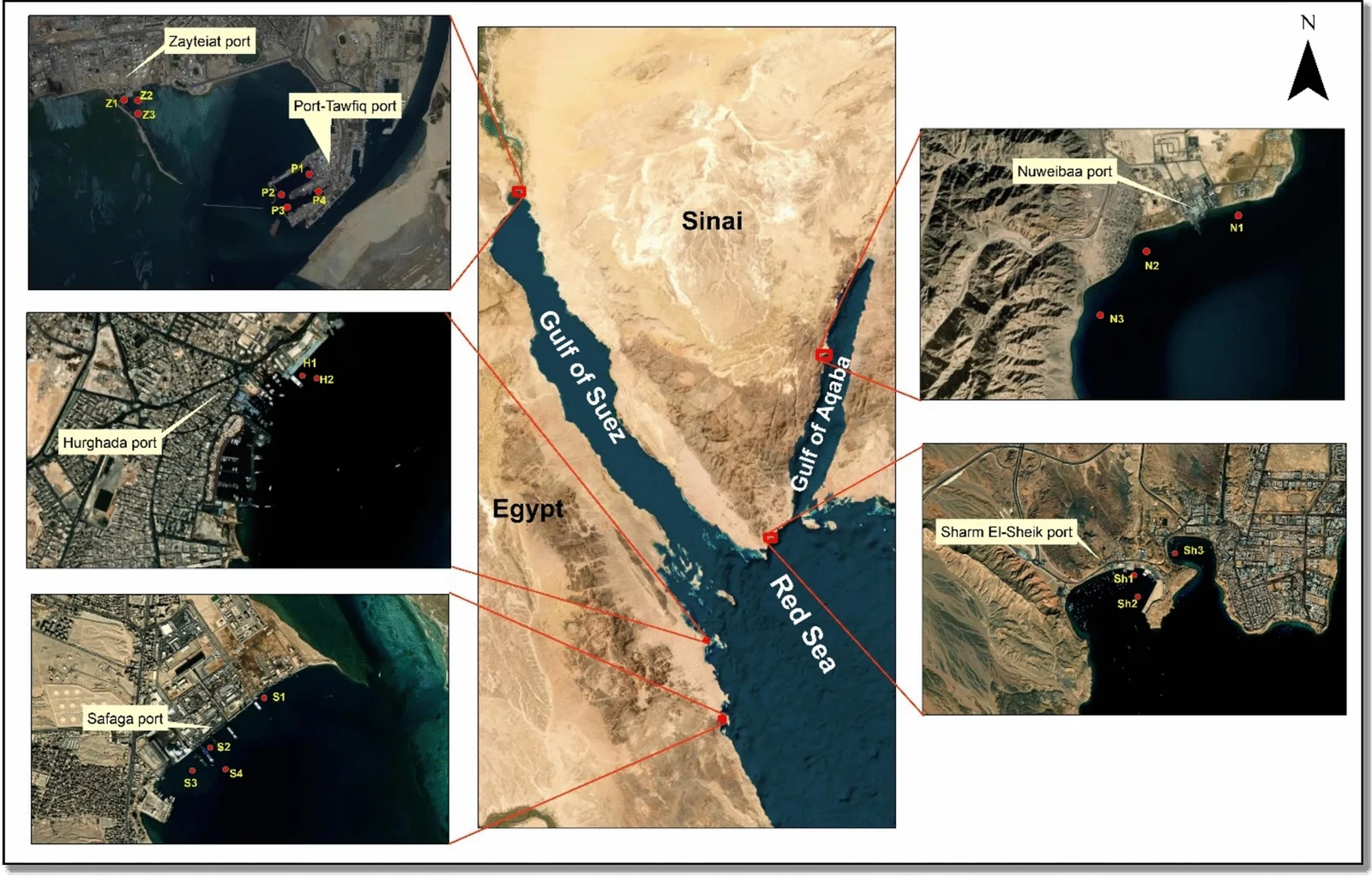
Location map of the study area along the Egyptian Red Sea coast, showing the six commercial and tourism ports investigated—Port-Tawfiq (P1–P4), Zayteiat (Z1–Z3), Hurghada (H1–H2), Safaga (S1–S4), Nuweibaa (N1–N3) and Sharm El-Sheikh (Sh1–Sh3)

Nineteen sampling stations were established across six commercial and tourism ports distributed along the Egyptian Red Sea coast (Fig. 1). Port-Tawfiq (stations P1–P4) anchors the northern Gulf of Suez at the southern entrance of the Suez Canal and combines passenger, container and ship-maintenance functions adjacent to the Suez Bay transit anchorage. Zayteiat (Z1–Z3) is a dedicated crude-oil terminal handling tanker discharge ~ 10 km southwest of Port-Tawfiq. Hurghada (H1–H2) serves international and yacht tourism on the western Red Sea coast and is bordered by extensive fringing coral reef systems. Safaga (S1–S4) is a phosphate-export terminal and pilgrim ferry gateway to Saudi Arabia, with an active Synchrolift ship-repair facility. Nuweibaa (N1–N3) operates daily car-ferry connections to the port of Aqaba (Jordan) on the Gulf of Aqaba coast. Sharm El-Sheikh (Sh1–Sh3), at the confluence of the Gulfs of Suez and Aqaba, functions primarily as a cruise terminal. Detailed environmental and operational profiles of each port are summarized in Soliman et al. (2010), Dar et al. (2016) and Mohamedein et al. (2023).

Surface seawater samples (0–0.5 m depth, 1 L per replicate, *n* = 2–4 per station per season) were collected with a 2.2 L acid-washed Niskin sampler. In total, 38 seawater samples and 19 sediment samples were analysed; seawater replication (*n* = 2–4 per station per season) varied among stations with accessibility and sea-state conditions during sampling. Sampling was conducted during the summer (July, 2017) and winter (January, 2018) seasons.

A single surficial sediment sample (0–5 cm) was collected from each station using a stainless-steel van Veen grab sampler (0.025 m^2^ jaw area). Seawater was transferred to pre-cleaned acid-washed (10% HNO_3_ for 24 h and rinsed three times with Milli-Q water) high-density polyethylene bottles, acidified in the field with concentrated trace-metal-grade HNO_3_ to pH < 2 and stored at 4 °C in the dark until analysis. Sediments were placed in pre-cleaned polypropylene containers, transported on ice, oven-dried at 60 °C to constant weight, gently disaggregated in an agate mortar and sieved through a 63 µm stainless-steel sieve; the fine (< 63 µm) fraction was retained for digestion because trace elements are preferentially associated with fine-grained sediments, in accordance with the recommended marine-sediment protocol (Loring & Rantala, 1992).

### Analytical procedures

Seawater sub-samples (10 mL) were pre-treated by acid oxidation: 0.5 mL of 30% HNO_3_ and 0.5 mL of 30% H_2_SO_4_ were added, followed by drop-wise 5% KMnO_4_ until a persistent violet colour confirmed complete oxidation, with a minimum 30 s contact time before measurement (Perkin-Elmer, 1978). Sediment sub-samples (0.500 g, accurately weighed, dry weight) were digested in a 95 °C water bath in a concentrated HNO_3_:H_2_SO_4_ mixture (3:1 v/v, 5 mL total) for 4 h, cooled to room temperature, diluted to 25 mL with Milli-Q water, and a 10-mL aliquot was processed using the same oxidative pre-treatment as for water (Adeloju et al., 1994); this hot concentrated-acid digestion yields pseudo-total, environmentally available rather than total, element concentrations.

Quantification of As, Se, Sn and Hg was performed on a Perkin-Elmer AAnalyst 100 atomic absorption spectrophotometer (Waltham, MA, USA) coupled to an MHS-10 hydride generation/cold-vapour system. Hydride generation (As, Se, Sn) used a 3% NaBH_4_/1% NaOH reductant; Hg was determined by cold-vapour atomic absorption (CV-AAS) using SnCl_2_ reduction. Calibration curves (*r*^2^ ≥ 0.998) were constructed from certified single-element 1000 mg L^−1^ stock standards (Merck, Darmstadt, Germany) diluted in matrix-matched blanks. Method detection limits, computed as three times the standard deviation of seven procedural blanks, were 0.10 µg L^−1^ (As, Se), 0.05 µg L^−1^ (Sn) and 0.02 µg L^−1^ (Hg) in water; 0.05 µg g^−1^ (As, Sn), 0.02 µg g^−1^ (Se) and 0.005 µg g^−1^ (Hg) in sediment. Method precision, assessed from replicate measurements (*n* = 5), ranged from 8.5 to 18.2% relative standard deviation depending on the element and concentration level, remaining within limits generally considered acceptable for environmental trace-element monitoring. Accuracy was verified against NIST SRM 1643e (trace elements in water) for the water matrix, with recoveries between 92 and 108%, and confirmed for sediments by matrix spike-recovery experiments (recovery 88–112%); a certified marine-sediment reference material was not available in our laboratory at the time of analysis, so spike recovery was used to verify sediment accuracy. All sediment concentrations are reported on a dry-weight basis.

### Sediment contamination indices

The contamination factor (CF), geoaccumulation index (I_geo_), pollution load index (PLI), metal pollution index (MPI), Hakanson ecological risk factor (E_r_) and potential ecological risk index (RI) were calculated following the established frameworks of Müller (1969), Håkanson (1980), Tomlinson et al. (1980) and Abrahim and Parker (2008).

#### Contamination factor

CF=Cm/Bm, where Cm is the measured sediment concentration (µg g^−1^) and Bm is the upper continental crust (UCC) background concentration. Because no local geochemical background values are currently available for the study area, two upper continental crust (UCC) reference datasets were applied in parallel to support sensitivity testing: Wedepohl (1995) [As 13.0, Se 0.6, Sn 6.0, Hg 1.4 µg g^−1^] and Rudnick and Gao (2003) [As 4.8, Se 0.09, Sn 2.1, Hg 0.05 µg g^−1^]; the Wedepohl (1995) baseline is used in the primary text for direct comparability with Mohamedein et al. (2023), El-Sorogy et al. (2020) and Nour et al. (2022). The Hakanson (1980) interpretive scheme classifies CF < 1 as low, 1 ≤ CF < 3 as moderate, 3 ≤ CF < 6 as considerable, and CF ≥ 6 as very high contamination.

#### Geoaccumulation index

$Igeo=log2\left(Cm/1.5\times Bm\right)$, with classification thresholds as defined by Müller (1969) and consistently applied by Khan et al. (2023) and El-Sorogy et al. (2024).

#### Pollution load index

$PLI=\left(CF1\times CF2\times \dots \times CFn\right)1/n$, with PLI > 1 indicating deterioration above background and PLI ≤ 1 indicating no station-scale pollution above baseline (Tomlinson et al., 1980).

#### Metal pollution index

$MPI=\left(C1\times C2\times \dots \times Cn\right)1/n$, where $C1\times C2\times \dots \times Cn$ is the multiplied concentrations of each individual heavy metal and n is the number of elements considered (Usero et al., 1997).

#### Hakanson ecological risk

$Er=Tr\times CF;RI=\Sigma Er$. The toxic-response factors used were T_r_ = 10 (As), 5 (Se, by analogy with Hakanson’s classification of moderately toxic metalloids in the absence of an original value; with results insensitive to the alternative T_r_ = 2), 2 (Sn) and 40 (Hg) (Håkanson, 1980; Yimer & Wepener, 2024). RI < 150 denotes low ecological risk; 150–300 moderate; 300–600 considerable; ≥ 600 very high.

### USEPA human health risk assessment

The chronic daily intake (CDI, mg kg^−1^ day^−1^) via oral ingestion of seawater (a conservative, upper-bound screening scenario for recreational swimmers, port workers and beach users) was estimated following USEPA () guidance:

$$CDI=\left(C\times IR\times EF\times ED\right)/\left(BW\times AT\right)$$

where C is the dissolved metal concentration (mg L^−1^; converted from µg L^−1^); IR is the ingestion rate, set here to the USEPA default drinking-water intake rates applied as a conservative upper-bound screening assumption (2.0 L day^−1^ for adults; 1.0 L day^−1^ for children); EF is the exposure frequency (365 day yr^−1^); ED is the exposure duration (30 yr adults; 6 yr children); BW is body weight (70 kg adults; 15 kg children); and AT is the averaging time (ED × 365 d for non-carcinogenic endpoints; 70 × 365 d for carcinogenic endpoints).

Non-carcinogenic risk was expressed as hazard quotient (HQ=CDI/RfD) and hazard index (HI=ΣHQ). The legacy USEPA oral reference doses (RfD, mg kg^−1^ day^−1^) were applied: As = 3.0 × 10^−4^; Se = 5.0 × 10^−3^; Sn = 6.0 × 10^−1^; Hg (inorganic) = 3.0 × 10^−4^ (USEPA IRIS, accessed 2024). The reassessed inorganic-As IRIS values issued by USEPA in 2025 (oral RfD 6.0 × 10^−5^ mg kg^−1^ day^−1^; oral cancer slope factor 32 mg^−1^ kg day) approximately five-fold more stringent for non-carcinogenic risk and ~ 21-fold more stringent for carcinogenic risk were not adopted in the primary computations to maintain comparability with the contemporaneous regional literature (El-Sorogy et al., 2024; Nour et al., 2022; Sayed et al., 2025); their implications are quantified in "Public-health implications: the children's HI exceedance and the IRIS-2025 sensitivity test" Section. Carcinogenic risk (CR) was estimated for As and Hg, the only Group 1/Group 2A carcinogens among the four elements, using oral cancer slope factors of SF_As_ = 1.5 × 10^−3^ (mg kg^−1^ day^−1^)^−1^ and SF_Hg_ = 1.0 × 10^−4^ (mg kg^−1^ day^−1^)^−1^. Acceptable lifetime CR is defined as 1 × 10^−6^ ≤ CR ≤ 1 × 10^−4^ (Eke, 2025; USEPA United States Environmental Protection Agency, 1989); CR < 1 × 10^−6^ is considered de minimis (ALARA). Carcinogenic risk was evaluated for the adult lifetime-exposure scenario, consistent with the 70-year averaging time used for cancer endpoints, which integrates exposure over the full lifespan; because a routinely applied USEPA IRIS oral slope factor for inorganic Hg is lacking, the Hg carcinogenic-risk estimate is treated as an indicative, screening-level value, and the carcinogenic-risk interpretation therefore focuses on As.

### Statistical analysis and multivariate methods

All statistical analyses were performed in Python 3.10 using NumPy 1.26, SciPy 1.11, scikit-learn 1.3 and pandas 2.1. Normality was assessed by Shapiro–Wilk test (α = 0.05) and homogeneity of variance by Levene’s test. Paired Student's t-tests compared summer and winter water concentrations across the 19 stations for each element; one-way ANOVA followed by Tukey HSD post-hoc tests compared annual port-level means. Pearson product–moment correlation matrices were computed for inter-element relationships in both water and sediment matrices, with significance evaluated at α = 0.05 and α = 0.01. Principal component analysis (PCA) was applied to the 19 stations, each described by the full set of eight seasonal water variables (As_S, Se_S, Sn_S, Hg_S, As_W, Se_W, Sn_W, Hg_W) after z-score standardisation; components with eigenvalues > 1 were retained according to the Kaiser criterion. Scree plots and component loadings are presented in Figs. S3 and S4.

## Results

### Trace element concentrations in seawater

Seasonal and annual mean concentrations (± SD) of dissolved As, Se, Sn and Hg across the six ports and two seasons are presented in Table 1; station-level data appear in Tables S1–S2 and Fig. S7. Across the full dataset (*n* = 38), concentrations spanned 2.197–12.451 µg L^−1^ for As; 0.104–0.580 µg L^−1^ for Se; 0.345–4.103 µg L^−1^ for Sn; and 0.083–0.583 µg L^−1^ for Hg.

**Table 1:** Annual mean (± SD) concentrations of dissolved trace elements in surface seawater (µg L^−1^) from six Egyptian Red Sea ports during summer 2017 and winter 2018, with seasonal grand means and USEPA water-quality criteria for comparison. *n* = 2–4 stations per port per season

Port	Season	As	Se	Sn	Hg
Port-Tawfiq	Summer	3.167 ± 0.876	0.357 ± 0.096	0.474 ± 0.127	0.115 ± 0.016
	Winter	10.536 ± 1.279	0.189 ± 0.025	2.179 ± 0.544	0.271 ± 0.110
Zayteiat	Summer	2.706 ± 0.767	0.506 ± 0.093	0.489 ± 0.025	0.109 ± 0.011
	Winter	9.634 ± 0.226	0.208 ± 0.069	2.607 ± 1.163	0.222 ± 0.087
Hurghada	Summer	2.556 ± 0.127	0.417 ± 0.054	0.582 ± 0.030	0.173 ± 0.027
	Winter	7.659 ± 0.210	0.143 ± 0.018	1.795 ± 0.544	0.208 ± 0.059
Safaga	Summer	2.607 ± 0.375	0.170 ± 0.049	0.474 ± 0.061	0.197 ± 0.024
	Winter	7.646 ± 0.437	0.150 ± 0.033	2.660 ± 1.222	0.365 ± 0.150
Nuweibaa	Summer	2.616 ± 0.301	0.313 ± 0.077	0.603 ± 0.129	0.160 ± 0.040
	Winter	9.107 ± 0.815	0.182 ± 0.052	1.282 ± 0.339	0.111 ± 0.048
Sharm El-Sheikh	Summer	2.750 ± 0.149	0.223 ± 0.089	0.747 ± 0.221	0.224 ± 0.040
	Winter	8.333 ± 0.318	0.182 ± 0.026	2.094 ± 0.783	0.167 ± 0.042
Grand mean	Summer	2.734	0.304	0.562	0.163
	Winter	8.819	0.176	2.103	0.224
USEPA criterion	–	36.0	71.0	–	0.94

**Fig. 2:**
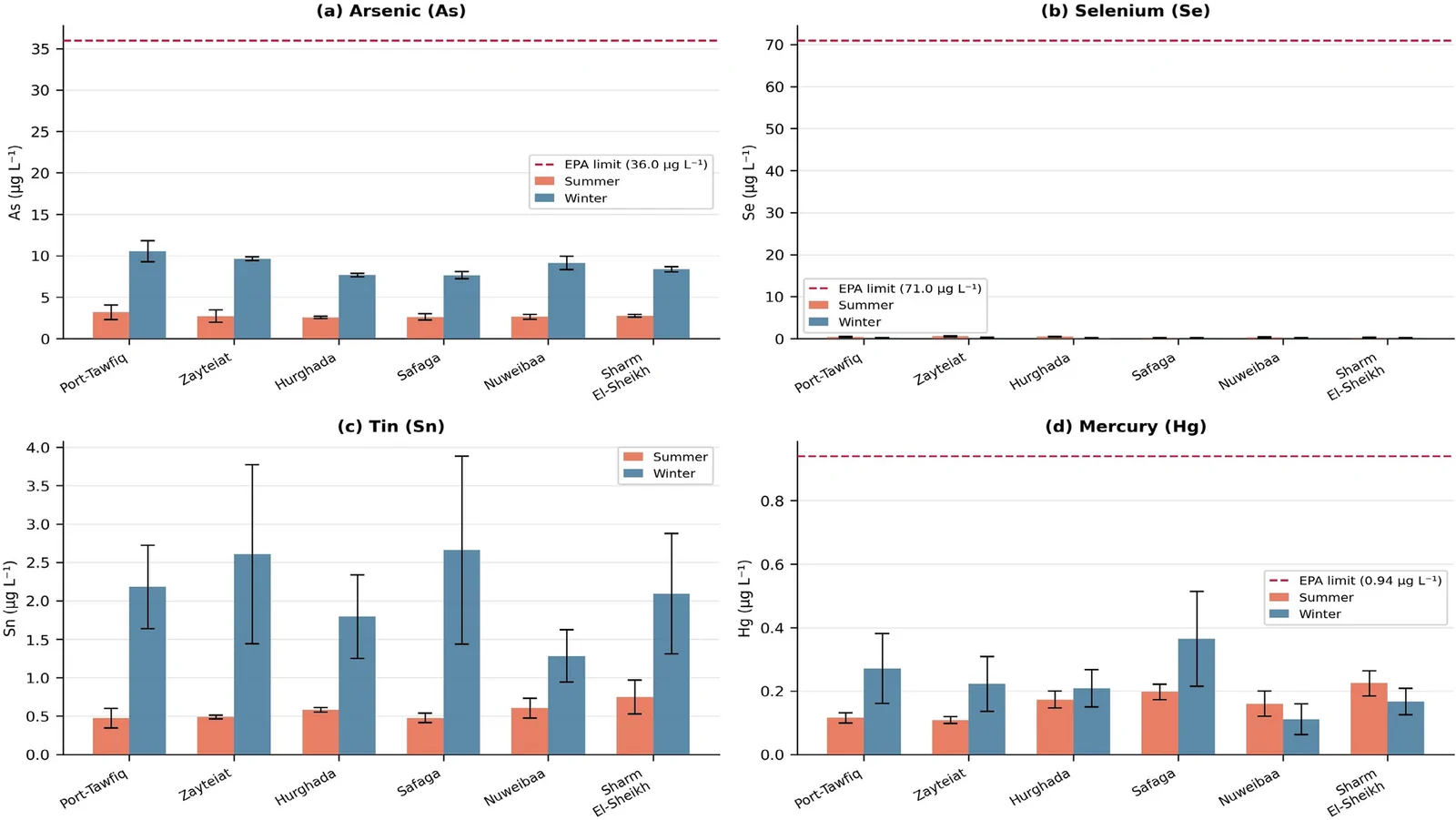
Seasonal distribution (mean ± SE) of **a** arsenic, **b** selenium, **c** tin and **d** mercury in surface seawater (µg L^−1^) from six Egyptian Red Sea ports during summer 2017 (orange) and winter 2018 (blue). Dashed red lines indicate USEPA water-quality criteria where applicable. Error bars represent ± 1 SE

Arsenic exhibited the strongest seasonal signal in the dataset. The overall winter mean (8.819 ± 1.041 µg L^−1^) was 3.23-fold higher than the overall summer mean (2.734 ± 0.526 µg L^−1^), a difference that was highly significant by paired t-test (t(18) = 20.08, *p* < 0.001; Fig. 2a, Fig. S1a). Maximum port-level winter mean As was recorded at Port-Tawfiq (10.536 ± 1.279 µg L^−1^); minimum was recorded at Hurghada in summer (2.556 ± 0.127 µg L^−1^). All 38 individual As values remained below the USEPA freshwater chronic criterion of 36 µg L^−1^, applied here in the absence of a corresponding marine chronic criterion (USEPA United States Environmental Protection Agency, 2024).

Selenium displayed the opposite seasonal pattern: summer means (0.319 ± 0.135 µg L^−1^) significantly exceeded winter means (0.177 ± 0.041 µg L^−1^; paired t(18) =  − 4.86, *p* < 0.001; Fig. 2b, Fig. S1b). The highest summer port mean for Se was recorded at Zayteiat (0.506 ± 0.093 µg L^−1^); all individual Se values (≈0.1–0.6 µg L^−^1) were well below any applicable water-quality criterion.

Tin concentrations were strongly elevated in winter relative to summer across all ports (overall summer mean 0.562 ± 0.131 µg L^−1^, winter mean 2.103 ± 0.766 µg L^−1^; winter values significantly exceeded summer values across all stations (paired *t*-test, *p* < 0.001); Fig. 2c). The highest port-level winter Sn was recorded at Safaga (2.660 ± 1.222 µg L^−1^), with significant within-port seasonal differences at Safaga (t(3) = 3.21, *p* = 0.012) and Sharm El-Sheikh (t(2) = 2.83, *p* = 0.046). Mercury concentrations were uniformly low; the highest port-level mean was recorded at Safaga in winter (0.365 ± 0.150 µg L^−1^), which remained below the USEPA water-quality criterion of 0.94 µg L^−1^ (Fig. 2d). One-way ANOVA on annual port means returned non-significant differences among the six ports for all four elements at α = 0.05 (As: F(5,33) = 0.26, *p* = 0.93; Se: F(5,33) = 2.48, *p* = 0.053; Sn: F(5,33) = 0.30, *p* = 0.91; Hg: F(5,33) = 1.98, *p* = 0.11), suggesting that seasonal variability was more pronounced than spatial variability in the seawater dataset.

### Trace element concentrations in surficial sediments

**Fig. 3:**
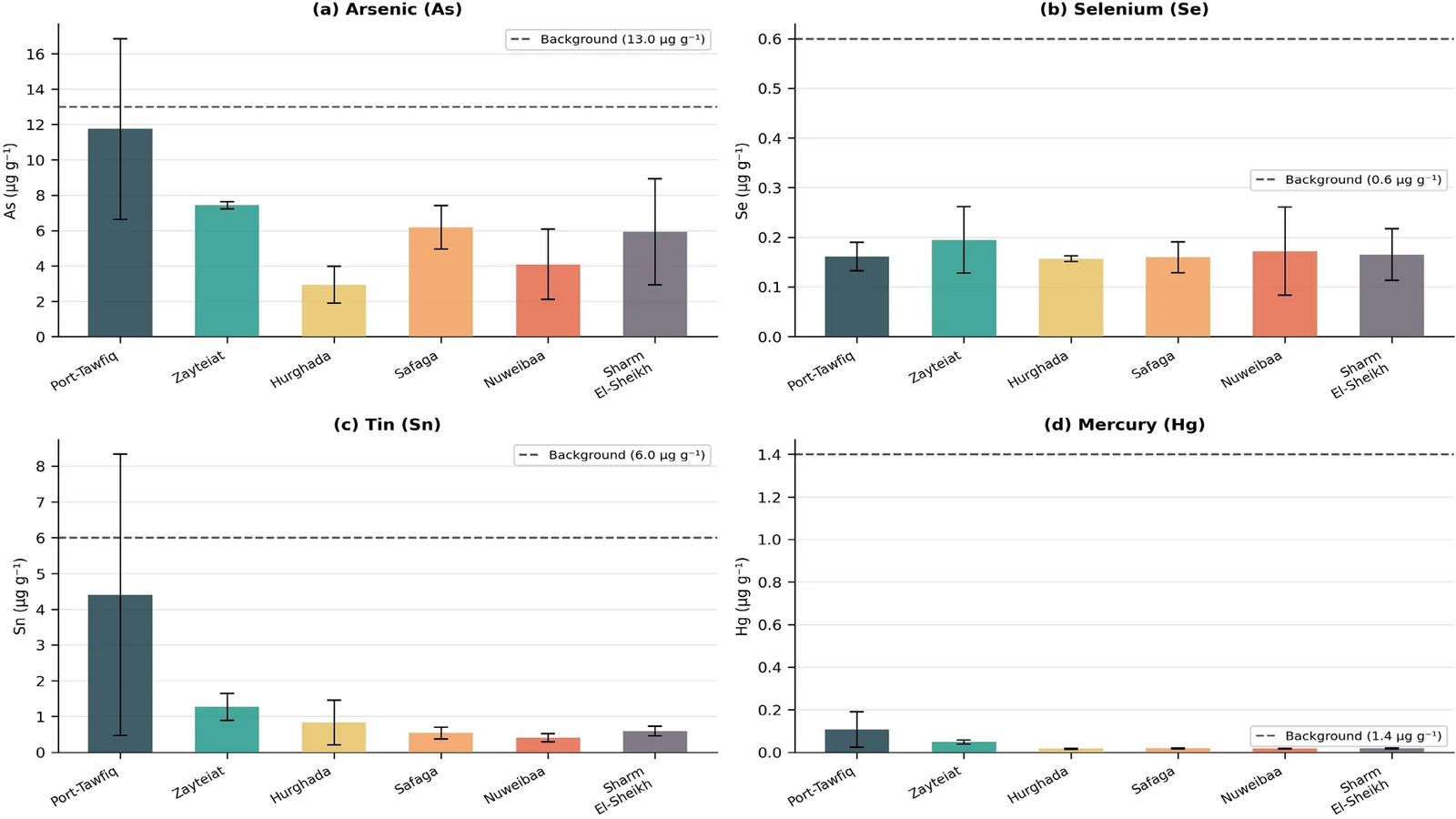
Mean concentrations (± SE) of **a** arsenic, **b** selenium, **c** tin and **d** mercury in surficial sediments (µg g^−1^) from six Egyptian Red Sea ports. Dashed grey lines indicate the upper continental crust background values of Wedepohl (1995)

Mean sediment concentrations of the four elements at the six ports, with global port and crustal background comparisons, are summarised in Table 2; station-level values are given in Table S3 and Fig. 3. Port-Tawfiq returned the highest mean sediment concentrations for As (11.748 ± 5.111 µg g^−1^), Sn (4.402 ± 3.931 µg g^−1^) and Hg (0.107 ± 0.084 µg g^−1^); Zayteiat returned the highest mean Se (0.195 ± 0.067 µg g^−1^). One-way ANOVA across the six ports yielded a significant difference for As only (F(5,13) = 3.72, *p* = 0.028); with Port-Tawfiq showing the highest mean sediment As; pairwise Tukey HSD comparisons for As are provided in the Supplementary Material. Differences for Se, Sn and Hg were non-significant (*p* > 0.05).

**Table 2:** Mean trace-element concentrations in surficial sediments (µg g^−1^) from six Egyptian Red Sea ports, with comparison to international port datasets and upper continental crust background values

Location	As	Se	Sn	Hg	Reference
Port-Tawfiq	11.75 ± 5.11	0.16 ± 0.03	4.40 ± 3.93	0.107 ± 0.084	This study
Zayteiat	7.43 ± 0.21	0.19 ± 0.07	1.27 ± 0.37	0.048 ± 0.009	This study
Hurghada	2.94 ± 1.05	0.16 ± 0.01	0.84 ± 0.62	0.017 ± 0.002	This study
Safaga	6.18 ± 1.24	0.16 ± 0.03	0.54 ± 0.16	0.019 ± 0.003	This study
Nuweibaa	4.09 ± 1.99	0.17 ± 0.09	0.41 ± 0.12	0.017 ± 0.002	This study
Sharm El-Sheikh	5.94 ± 3.00	0.17 ± 0.05	0.59 ± 0.13	0.018 ± 0.002	This study
Damietta, Egypt	7.40	–	–	0.100	El-Gohary et al. (2017)
Port Klang, Malaysia	34.1–112.8	–	–	0.17–0.35	Tavakoly Sany et al. (2012)
S. Korean harbours	4.2–11.9	–	–	0.0004–0.094	Choi et al. (2012)
Port Kembla, Australia	46–340	< 5–140	–	–	Jones et al. (2019)
Background (UCC)	13.0	0.60	6.0	1.4	Wedepohl (1995)
Background (UCC)	4.8	0.09	2.1	0.05	Rudnick and Gao (2003)

All port-level mean concentrations remained below the upper continental crust background of Wedepohl (1995) (As 13.0; Se 0.6; Sn 6.0; Hg 1.4 µg g^−1^). At the individual-station level, however, As at Port-Tawfiq station P3 reached 17.50 µg g^−1^—1.35 t the Wedepohl background value, and Sn at P1 reached 8.63 µg g^−1^ (1.44 t background). Hg never exceeded 0.20 µg g^−1^ at any station (maximum 0.198 µg g^−1^ at P3, i.e. 14% of background).

### Sediment contamination indices

**Fig. 4:**
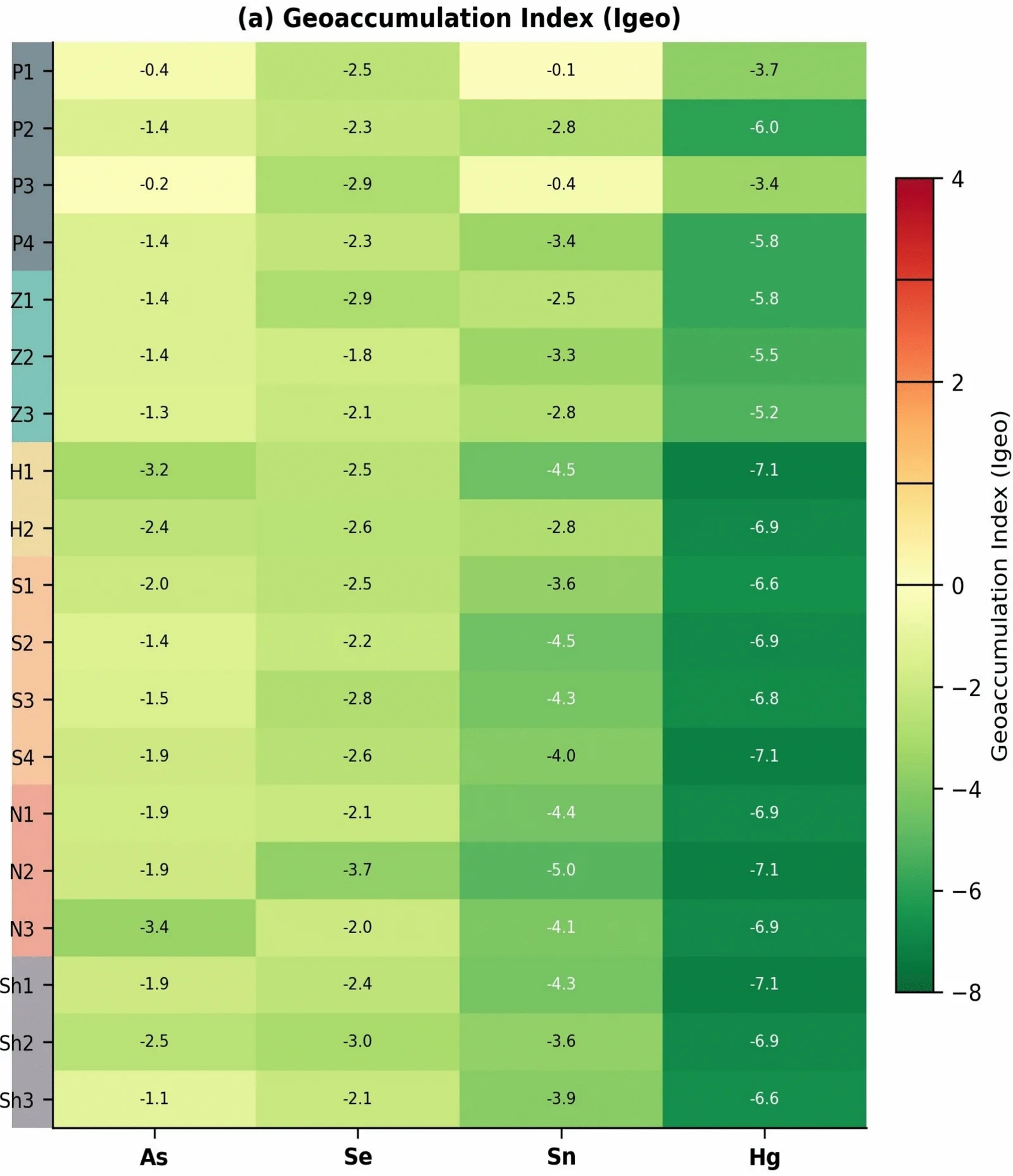

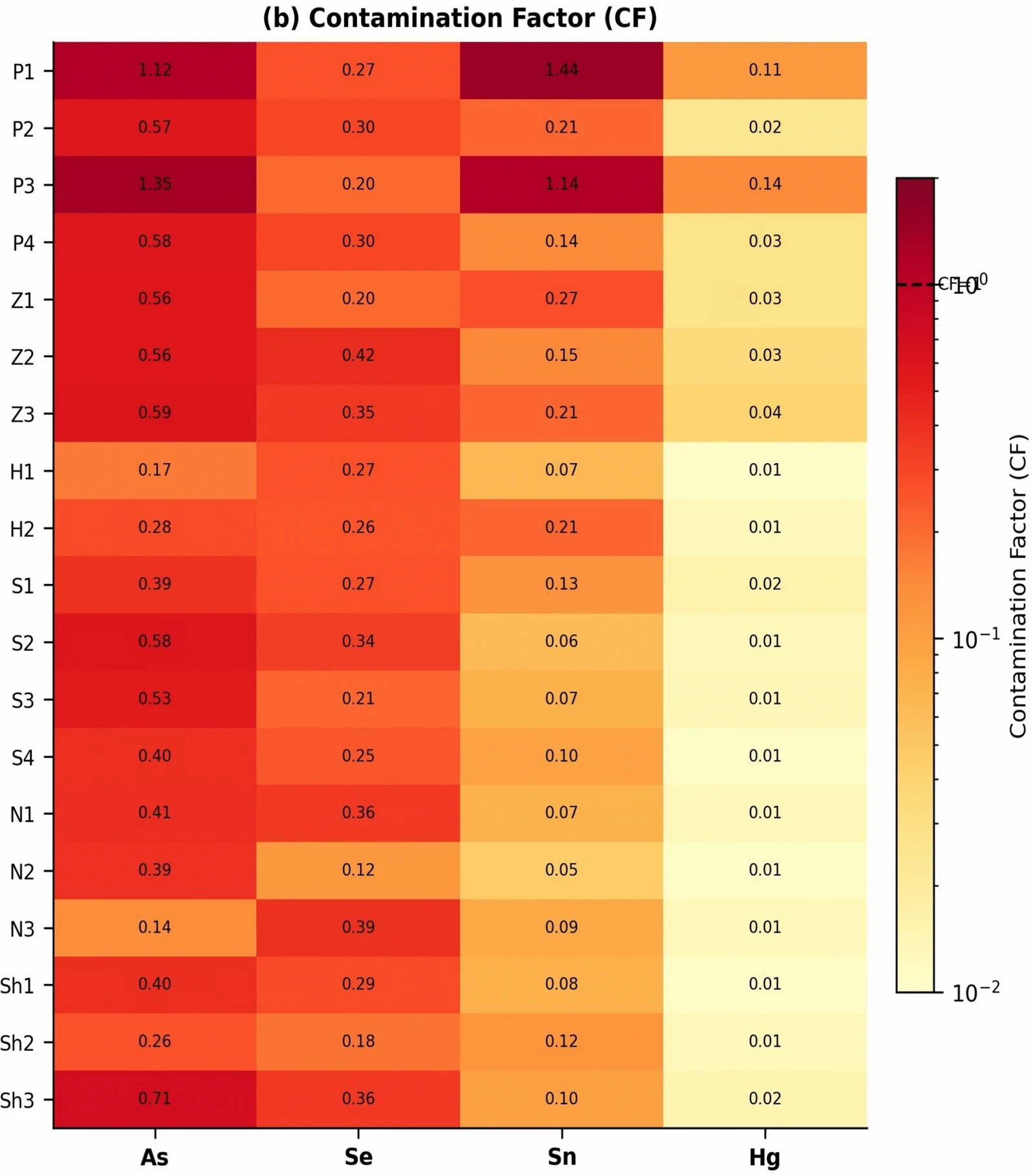
Dual heatmaps of **a** geoaccumulation index (I_geo_) and **b** contamination factor (CF) for As, Se, Sn and Hg at the 19 sediment stations. The CF panel uses a logarithmic colour scale to visualise the wide dynamic range; the dashed line denotes CF = 1

**Fig. 5:**
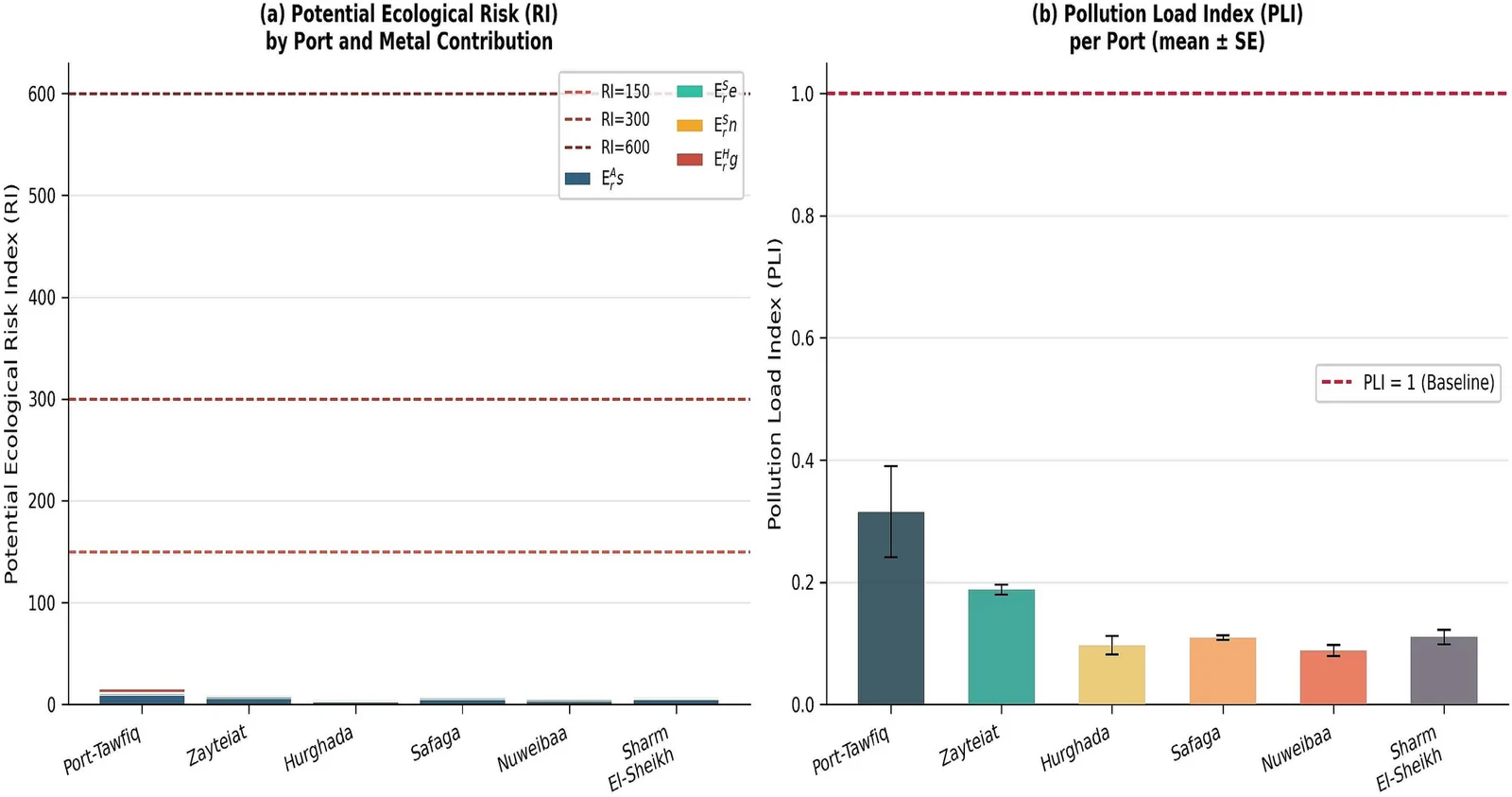
**a** Stacked potential ecological risk index (RI) by port and individual metal contribution (E_r_); dashed horizontal lines denote RI = 150 (low/moderate threshold), 300 (moderate/considerable) and 600 (considerable/very high). **b** Pollution load index (PLI) per port (mean ± SE); dashed red line indicates PLI = 1 baseline threshold

Station-level CF, I_geo_, PLI and RI values are presented in Table 3 and graphically in Fig. 4,5 and Fig. S5. CF values for As ranged from 0.14 (N3, Nuweibaa) to 1.35 (P3, Port-Tawfiq), placing only two stations (P1, CF = 1.12; P3, CF = 1.35) in the moderate-contamination class. CF values for Se (0.12–0.42) and Hg (0.01–0.14) were uniformly < 1, whereas Sn ranged from 0.05 to 1.44, with only station P1 exceeding unity.

**Table 3:** Station-level contamination factor (CF) for each metal, pollution load index (PLI), Hakanson potential ecological risk index (RI) and risk class for the 19 sediment stations. Background values follow Wedepohl (1995)

Station	Port	CF_As	CF_Se	CF_Sn	CF_Hg	PLI	RI	Class
P1	Port-Tawfiq	1.124	0.268	1.439	0.113	0.470	19.97	Low
P2	Port-Tawfiq	0.569	0.303	0.211	0.024	0.171	8.57	Low
P3	Port-Tawfiq	1.346	0.202	1.143	0.141	0.458	22.41	Low
P4	Port-Tawfiq	0.576	0.303	0.142	0.028	0.162	8.68	Low
Z1	Zayteiat	0.564	0.202	0.272	0.028	0.171	8.31	Low
Z2	Zayteiat	0.561	0.420	0.147	0.034	0.186	9.38	Low
Z3	Zayteiat	0.590	0.352	0.214	0.041	0.206	9.71	Low
H1	Hurghada	0.169	0.268	0.066	0.011	0.075	3.59	Low
H2	Hurghada	0.283	0.255	0.212	0.013	0.118	5.04	Low
S1	Safaga	0.387	0.267	0.126	0.016	0.120	6.09	Low
S2	Safaga	0.584	0.335	0.064	0.013	0.113	8.16	Low
S3	Safaga	0.525	0.212	0.074	0.014	0.103	7.00	Low
S4	Safaga	0.405	0.252	0.096	0.011	0.101	5.93	Low
N1	Nuweibaa	0.411	0.357	0.071	0.013	0.108	6.56	Low
N2	Nuweibaa	0.393	0.117	0.047	0.011	0.069	5.04	Low
N3	Nuweibaa	0.138	0.387	0.085	0.013	0.087	4.00	Low
Sh1	Sharm El-Sheikh	0.402	0.285	0.075	0.011	0.098	6.02	Low
Sh2	Sharm El-Sheikh	0.258	0.185	0.120	0.013	0.093	4.26	Low
Sh3	Sharm El-Sheikh	0.710	0.357	0.101	0.015	0.140	9.68	Low

Geoaccumulation indices were negative for all stations and all elements. Although As exhibited the highest I_geo_ values, at P1, P3 and P4 (− 0.4, − 0.2 and − 1.4, respectively), all values remained within the uncontaminated category. Maximum I_geo_ across the dataset was − 0.1 for Sn at P1 (Table 3). PLI values ranged from 0.069 (N2) to 0.470 (P1) and were uniformly < 1 (Table 3). The potential ecological risk index (RI) ranged from 3.59 (H1) to 22.41 (P3), well below the threshold for moderate ecological risk (RI = 150; Håkanson, 1980), placing every station in the “low ecological risk” class. Across the 19 stations, As contributed on average 67% of total RI, Hg contributed 27%, Se contributed 5% and Sn contributed 1%, reflecting the combined effect of contamination magnitude and toxic-response factor.

### Human health risk via oral seawater ingestion

**Fig. 6:**
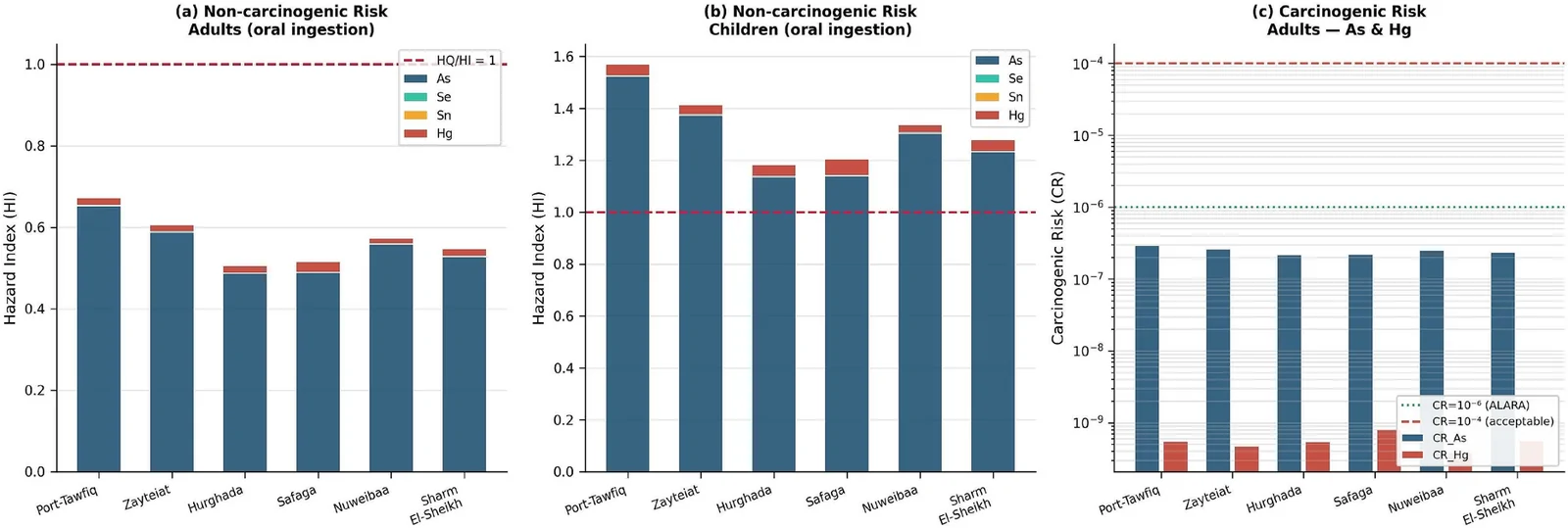
USEPA human health risk assessment via oral seawater ingestion across the six ports: **a** non-carcinogenic hazard index for adults (stacked by metal contribution); **b** non-carcinogenic hazard index for children (note y-axis exceeds the threshold of 1 at every port); **c** carcinogenic risk (CR) for As and Hg for adults, on a logarithmic scale, with USEPA reference thresholds CR = 10^−6^ (ALARA) and 10^−4^ (acceptable maximum)

Hazard quotients (HQ) for each element, total hazard index (HI) for adults and children, and total carcinogenic risk (TCR) for adults via oral seawater ingestion are summarised in Table 4 and Figs. 6 and S6. For adults, port-level HI ranged from 0.497 (Hurghada) to 0.671 (Port-Tawfiq), all below the threshold value of 1. Arsenic contributed 84–97% of total adult HI across the six ports; mercury contributed 2–4%; selenium and tin together contributed < 1%.

**Table 4:** Non-carcinogenic hazard quotient (HQ) per element, hazard index (HI) for adults and children, and total carcinogenic risk (TCR) for adults via oral seawater ingestion at the six Egyptian Red Sea ports. HI values exceeding the safety threshold of 1 are highlighted in bold

Port	HQ_As	HQ_Se	HQ_Sn	HQ_Hg	HI (adult)	HI (child)	TCR (adult)
Port-Tawfiq	0.651	0.002	< 0.001	0.018	0.671	1.566	4.60 × 10^−5^
Zayteiat	0.583	0.002	< 0.001	0.016	0.601	1.413	4.14 × 10^−5^
Hurghada	0.481	0.002	< 0.001	0.014	0.497	1.131	3.43 × 10^−5^
Safaga	0.485	0.001	< 0.001	0.016	0.502	1.198	3.45 × 10^−5^
Nuweibaa	0.555	0.001	< 0.001	0.013	0.569	1.328	3.94 × 10^−5^
Sharm El-Sheikh	0.524	0.001	< 0.001	0.014	0.539	1.265	3.72 × 10^−5^

For children, port-level HI ranged from 1.131 (Hurghada) to 1.566 (Port-Tawfiq); all six ports exceeded the safety threshold (Fig. 6b, Fig. S6b). Decomposition of the children’s HI showed that As accounted for 91.6–97.0% of the total at each port. Among the remaining ports, HI values decreased in the order Zayteiat (1.413), Nuweibaa (1.328), Sharm El-Sheikh (1.265), Safaga (1.198) and Hurghada (1.131). The HQ_As component alone exceeded 1.0 at Port-Tawfiq (1.519), Zayteiat (1.370) and Nuweibaa (1.296), and ranged 1.13–1.22 at the remaining three ports.

Total carcinogenic risk for adults ranged from 3.43 × 10^−5^ (Hurghada) to 4.60 × 10^−5^ (Port-Tawfiq) and lay within the USEPA acceptable range (10^−6^ to 10^−4^; Fig. 6c). The mercury contribution to TCR was < 0.3% at every port; arsenic therefore accounted for more than 99% of total carcinogenic risk at all ports.

### Correlation structure and principal component analysis

**Fig. 7:**
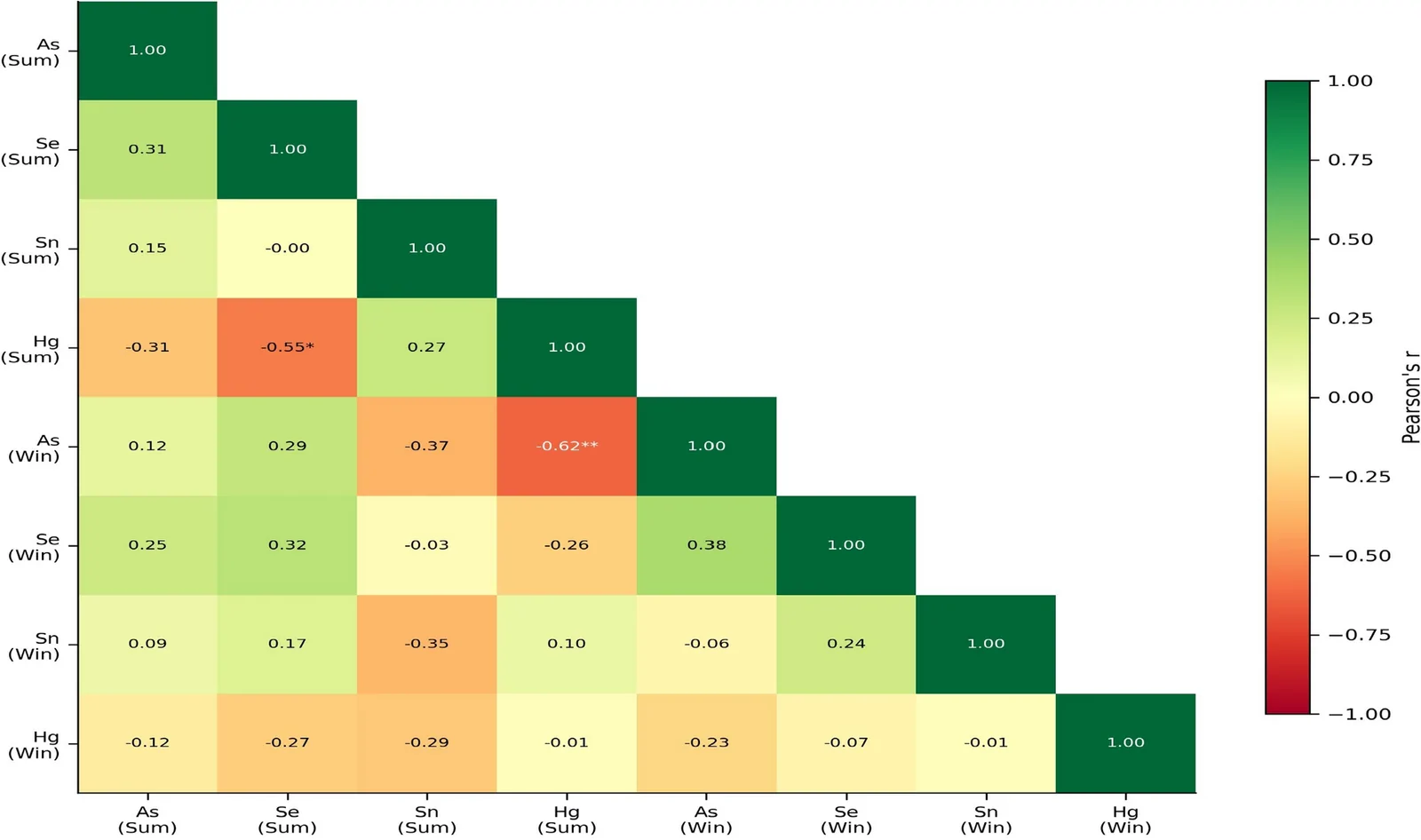
Pearson correlation matrix of seasonal trace-element concentrations in seawater across the 19 stations. (Sum) = summer 2017, (Win) = winter 2018. *p < 0.05; **p < 0.01

Pearson correlation matrices for sediment and seasonal seawater variables are provided in Tables S7 and Fig. 7. In sediments, strong, highly significant positive correlations were detected among As, Sn and Hg: r(As–Sn) = 0.837 (*p* < 0.001), r(As–Hg) = 0.899 (*p* < 0.001) and r(Sn–Hg) = 0.949 (*p* < 0.001) (Fig. S2). Selenium showed no significant correlations with other elements in sediments (|r|≤ 0.18).

In seawater, the dominant correlations were a significant negative correlation between summer Se and summer Hg (*r* =  − 0.55, *p* < 0.05) and a significant negative correlation between summer Hg and winter As (*r* =  − 0.62, *p* < 0.01) (Fig. 7). All other inter-element correlations in the seasonal water dataset were non-significant at α = 0.05.

**Fig. 8:**
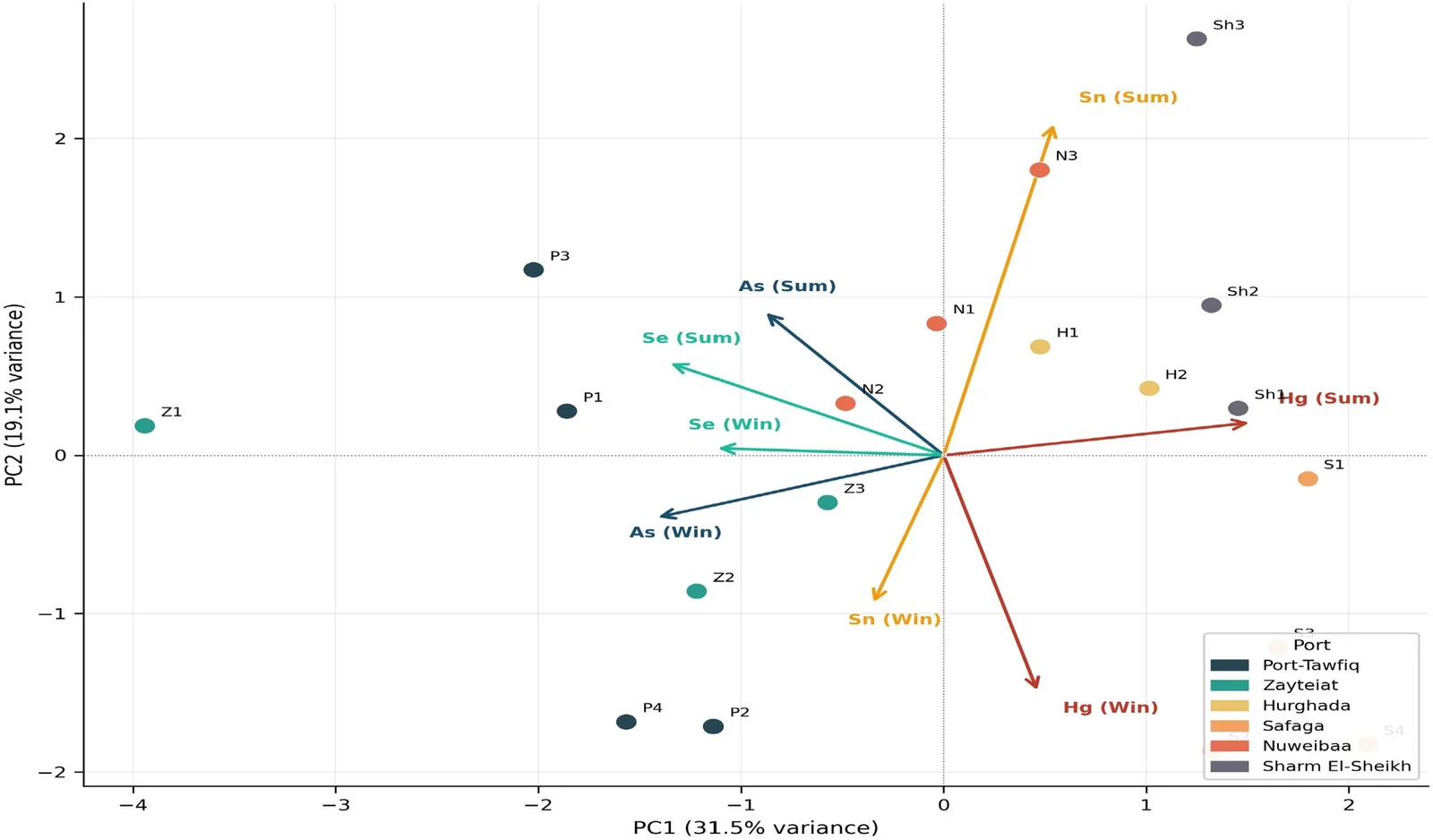
Principal component analysis biplot of seasonal trace-element variability in seawater across the 19 stations. Arrows are the PC loading vectors of the eight seasonal variables (As, Se, Sn, Hg in summer and winter, abbreviated Sum and Win). Symbols are station scores coloured by port. PC1 explains 31.5% and PC2 explains 19.1% of the total variance

Principal component analysis of the eight seasonal water variables (Fig. 8, Figs. S3–S4) extracted two components with eigenvalues > 1, explaining 31.5% (PC1) and 19.1% (PC2) of total variance (cumulative 50.6%); four additional components were required to explain 80% of the variance (Fig. S3). PC1 loaded negatively on summer Se (− 0.477), summer Sn (− 0.456), summer Hg (− 0.380) and summer As (− 0.298), and positively on winter Sn (+ 0.511) and winter As (+ 0.183), representing a seasonal contrast between summer and winter conditions. PC2 loaded positively on winter As (+ 0.703) and summer As (+ 0.305) and negatively on winter Hg (− 0.501) and winter Se (− 0.316), associated with contrasting winter As and winter Hg patterns. In the PC1–PC2 score space (Fig. 8), Port-Tawfiq stations (P1–P4) and Zayteiat stations (Z1–Z3) grouped towards the negative PC1 region, Safaga (S1–S4) and Sharm El-Sheikh (Sh1–Sh3) towards the positive PC1 region, and Hurghada (H1–H2) and Nuweibaa (N1–N3) near the centroid of the score plot.

### Integrated multi-index assessment

**Fig. 9:**
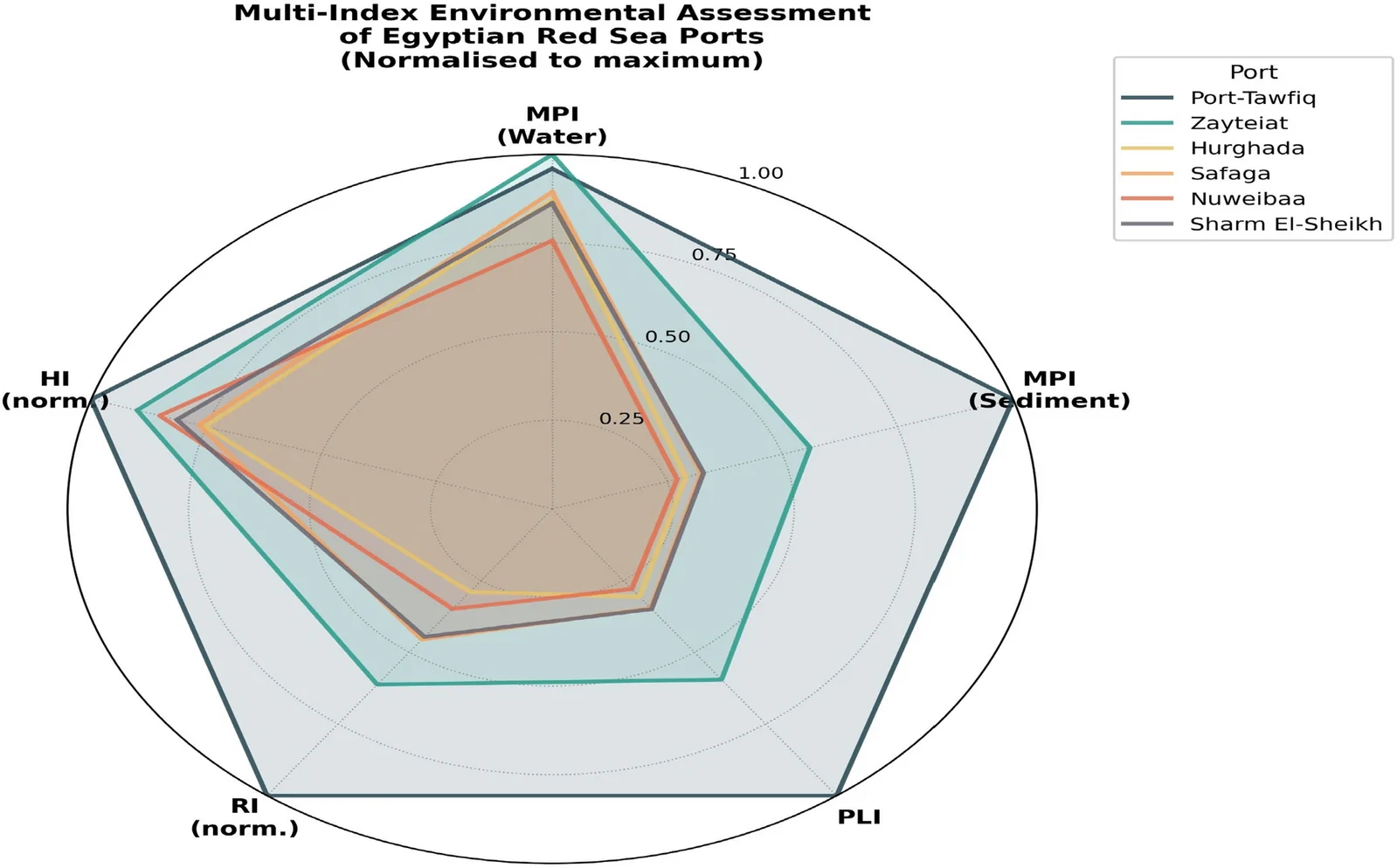
Multi-index radar diagram comparing the six ports across five normalised indicators (MPI_water_, MPI_sediment_, PLI, RI_norm_, HI_norm_). Each axis is normalised to the maximum value across the six ports

To synthesize the five multi-index results across the six ports, normalised values of MPI_water_, MPI_sediment_, PLI, RI and HI (adult) are presented as a radar diagram in Fig. 9. Port-Tawfiq returned the largest polygon area across all five normalised indicators, followed by Zayteiat (highest on MPI_water_ and MPI_sediment_). Hurghada, Safaga, Nuweibaa and Sharm El-Sheikh showed largely overlapping and similar-sized polygons, with Safaga showing a slightly higher PLI compared with the other tourism and ferry ports.

## Discussion

### Seasonal biogeochemical drivers of dissolved As, Se, Sn and Hg

The 3.2-fold winter enrichment of dissolved As observed across all six Egyptian Red Sea ports is mechanistically coherent with two well-documented marine biogeochemical processes. First, arsenate (AsV) is a structural and chemical analogue of phosphate and is taken up by phytoplankton via the same transmembrane transport systems, after which marine microbiota actively detoxify it through methylation to dimethylarsenate (DMA) and arsenosugars, followed by transfer through the marine food web and eventual release to the dissolved phase (Khan et al., 2023; Smedley & Kinniburgh, 2002; Wang et al., 2021). In Egyptian Red Sea waters, summer phytoplankton biomass is documented as substantially higher than winter biomass (Nassar et al., 2016), and the resulting summer drawdown of dissolved AsV combined with the loss of methylated As species to the atmosphere via dimethylarsine evasion under elevated sea-surface temperatures (Riedel, 1993). Together, these processes produce the summer minimum observed here. Yu et al. (2025) recently demonstrated experimentally that AsV biotransformation rates in marine macroalgae double under high-salinity, warm-temperate conditions, providing direct empirical support for our summer-depletion interpretation in a hypersaline (~ 40 PSU) system. The mechanism converges with seasonal patterns reported by Khan et al. (2023) for Atlantic and Pacific surface waters, but its expression in the Red Sea is amplified by the basin’s exceptional evaporation regime and limited dilution capacity.

The inverse seasonality of Se (summer maximum, winter minimum) is consistent with two co-acting drivers. First, salinity-controlled partitioning of selenite/selenate towards the dissolved phase intensifies under the high-evaporation, high-irradiance summer conditions characteristic of the northern Red Sea (Breuninger et al., 2023; Sugimura et al., 1976). Second, marine phytoplankton transform inorganic Se to organic species (selenoamino acids, selenium-containing methylated metabolites) that are released to the water column upon cell lysis, with intracellular Se speciation patterns now resolved at species level by LC-ICP-MS/MS (Mihaljevič et al., 2024). The fact that the highest summer Se mean was recorded at Zayteiat (0.506 µg L^−1^) an oil terminal handling crude petroleum naturally enriched in organoselenium provides a second independent source mechanism: hydrocarbon-bound Se progressively partitions into solution during loading/unloading and minor spills, and is preferentially retained dissolved in summer when surface evasion is suppressed by high salinity (Lemly, 2002). Selenium's independence from the As–Sn–Hg correlation cluster in the sediment dataset ("Correlation structure and principal component analysis" Section) reinforces this dual interpretation: Se appears to be influenced by petroleum-related inputs more than antifouling-paint sources.

The strong winter Sn enrichment, most pronounced at Safaga (2.660 µg L^−1^) and Zayteiat (2.607 µg L^−1^), is best explained by the temperature-dependent persistence of tributyltin (TBT) and its degradation intermediates. The Agency for Toxic Substances and Disease Registry reports TBT seawater half-lives ranging from approximately 6 days in particle-rich, warm summer conditions to 127 days in clean, cold winter water (Agency for Toxic Substances & Disease Registry)., 2005). Reduced winter solar irradiance further attenuates the principal degradation pathway direct UV photolysis and OH-radical mediated photodegradation (Rodriguez-Gonzalez et al., 2013) allowing TBT inputs from antifouling-paint leachate during vessel idling, hull cleaning and dry-dock operations to accumulate. Safaga’s dual role as a high-traffic phosphate/passenger port with an active Synchrolift ship-repair yard makes it a plausible focal point for organotin loading, consistent with the elevated Cu, Zn and Sn signal previously documented in the adjacent nearshore zone by Dar et al. (2016). Critically, the observation of winter Sn enrichment (more than a decade after the 2008 entry-into-force of the IMO AFS Convention banning organotin biocides on ship hulls; IMO International Maritime Organization. 2008. International Convention on the Control of Harmful Anti-Fouling Systems on Ships (AFS Convention). Adopted 5, 2008) aligns with recent post-ban evidence from the Baltic Sea (Bełdowska et al., 2023) and the South Atlantic Nuevo Gulf (Nudi et al., 2024), where butyltin signals persist in sediments and the water column despite international prohibition. Whether the residual signal originates from vessels operating under non-compliant flags, from undocumented repainting in informal yards, or from legacy paint particles in port sediments and dredge spoils cannot be resolved from total-Sn data alone; speciation-resolved butyltin analysis is therefore an essential next step.

Mercury’s winter enrichment at Port-Tawfiq, Zayteiat, Safaga and Hurghada (Fig. 2d) is mechanistically consistent with the inverse temperature dependence of Hg^0^ volatilisation across the air–sea interface: cooler winter surface temperatures suppress Hg evasion and retain dissolved Hg in the water column (Wang et al., 2023; Zhou & Zhang, 2024). Modelling work by Wang et al. (2023) projects that ongoing surface-ocean warming will further perturb the marine Hg cycle, reducing methylmercury concentrations by ~ 6% globally by 2100 but with strong regional variability dependent on coastal productivity and DOM dynamics. The observation that summer Hg maxima at Sharm El-Sheikh (0.224 µg L^−1^) and Safaga (0.197 µg L^−1^) coincide with peak tourism and recreational boating intensity also suggests a possible anthropogenic input pathway (small-vessel paint, fuel and bilge discharges) that is plausibly distinct from the temperature-mediated winter signal at the cargo-and-tanker ports. Future studies in this corridor should pair total Hg with methylmercury and reactive Hg(II) speciation to disentangle these overlapping forcings.

### Source apportionment: an antifouling-paint legacy signal and a petroleum signal

The strong, statistically robust positive correlations among As, Sn and Hg in surficial sediments (*r* ≥ 0.84, *p* < 0.001 in all pairs; Fig. S2) and the co-elevation of these three elements at Port-Tawfiq stations P1 and P3 strongly suggest antifouling-paint residues as a major anthropogenic source in this system. Marine antifouling formulations historically included organotins (TBT, DBT) as the primary biocide and, in some formulations, additional biocidal compounds including As- and Hg-bearing additives (Lagerström et al., 2020; Sarkar, 2018). Although TBT was banned under the 2008 AFS Convention, the legacy paint matrix (abraded from hulls during vessel transit, idling and maintenance) continues to release these co-occurring biocides to port sediments, where their joint deposition produces precisely the inter-element correlation pattern observed here (Bełdowska et al., 2023; Nudi et al., 2024). The Port-Tawfiq P3 station, with sediment As at 17.50 µg g^−1^, Sn at 6.86 µg g^−1^ and Hg at 0.198 µg g^−1^, represents a localised antifouling-paint hotspot consistent with prolonged vessel idling at the Suez Bay transit anchorage (Soliman et al., 2010). The complementary PCA result, in which Port-Tawfiq and Zayteiat stations cluster in the negative PC1 quadrant alongside summer Sn, Se and Hg loadings (Fig. 8), supports this attribution from an independent multivariate perspective.

Selenium occupies a distinct source niche. Its statistical independence from the As–Sn–Hg cluster in both the sediment correlation matrix (|r|≤ 0.18, all *p* > 0.45) and the seawater PCA loading structure (Fig. 8), combined with its highest concentrations at Zayteiat in both seawater and sediment, supports petroleum operations as the primary local Se source. Crude oils can contain Se concentrations elevated relative to background crustal materials, reflecting their formation from marine organic matter (Lemly, 2002; Schintu et al., 2016). The co-enrichment of Se with petroleum-derived hydrocarbons at oil terminals is well documented in the Persian Gulf (Bagheri et al., 2024), where similar tanker-handling operations produce a localised, port-specific Se signal that mirrors the pattern observed here at Zayteiat.

Across the tourism and ferry ports (Hurghada, Nuweibaa, Sharm El-Sheikh), all sediment contamination indices remained below their critical thresholds, with CF < 1 for all elements, PLI between 0.069 and 0.320, and RI between 3.6 and 9.7 at every station. Sharm El-Sheikh's elevated summer Sn and Hg signal in water, however, may reflect intensified small-vessel and recreational-boat traffic during the peak tourism season, consistent with the photo-degradation/temperature model described in "Seasonal biogeochemical drivers of dissolved As, Se, Sn and Hg" Section and with antifouling-paint inputs from leisure craft documented at recreational boatyards globally (Lagerström et al., 2016). Taken together, the contamination gradient Port-Tawfiq > Zayteiat > Safaga > Sharm El-Sheikh ≈ Hurghada ≈ Nuweibaa reflects a decreasing intensity of heavy shipping and industrial activity, matching the operational classification of the six ports independently and reproducing the MPI rankings of Mohamedein et al. (2023) for both compartments. The convergence of multiple, methodologically distinct lines of evidence CF, I_geo_, PLI, RI, PCA and bivariate correlations on the same source structure provides strong support for the source attribution and addresses the methodological gap.

### Regional and global benchmarking of port sediment contamination

Placed against international port datasets (Table 2), Egyptian Red Sea sediment As concentrations (used here as a representative trace element) are broadly comparable to South Korean harbours (4.2–11.9 µg g^−1^; Choi et al., 2012) and Damietta, Egypt (7.40 µg g^−1^; El-Gohary et al., 2017), but are an order of magnitude lower than the heavily industrialised Port Kembla, Australia (46–340 µg g^−1^; Jones et al., 2019) and Port Klang, Malaysia (34.1–112.8 µg g^−1^; Tavakoly Sany et al., 2012). The RI < 30 maximum observed across our 19 stations is consistent with recent assessments of the Hurghada coast (El-Sorogy et al., 2024), the Gulf of Suez coastal zone (El-Sawy et al., 2023; Nour et al., 2022) and the Saudi Red Sea–Gulf of Aqaba coast (El-Sorogy et al., 2020), which collectively place the Egyptian Red Sea in a moderate-to-low contamination class relative to industrialised East Asian and European port systems. The persistence of this relatively low contamination level despite high shipping density may reflect the combination of an exceptionally low terrestrial freshwater input (the Red Sea has no perennial river inflow and the high carbonate sediment background characteristic of the region) which may reduce the availability of fine-grained binding phases for trace metals (Mohamedein et al., 2023).

Two recent regional studies provide particularly close comparators. Elgendy et al. (2024), in their NIOF-led assessment of El-Quseir and Safaga coastal sediments, reported CF values for As, Cd, Pb and Zn that are broadly consistent with the present dataset (predominantly CF < 1; isolated CF ≥ 1 for selected metals at specific stations) and identified maritime traffic and ship-maintenance activities as the principal anthropogenic source, providing independent corroboration of our source-apportionment conclusion. El-Sorogy et al. (2024) similarly classified Hurghada nearshore sediments as moderately Pb-polluted but unpolluted to lightly polluted for the remaining metals examined, with adult and child hazard indices below 1; the present study extends that picture by showing that the children’s HI exceeds 1 when arsenic is included in the assessed metal suite, a result that previous Egyptian Red Sea analyses have not reported. Beyond Egypt, Bagheri et al. (2024) and the Persian Gulf meta-analysis of Niri et al. (2025) place the Arabian Peninsula's port systems in a moderately contaminated regime; our Egyptian Red Sea values appear comparatively lower for As, Sn and Hg, although the Persian Gulf contamination signal is often dominated by Pb, Ni and V, which are not the focus of the present study.

### Public-health implications: the children's HI exceedance and the IRIS-2025 sensitivity test

The most policy-relevant finding of the present study is the children’s hazard index exceeding 1 at all six ports (HI = 1.131–1.566), driven by arsenic (HQ_As contributes 91.6–97.0% of the total HI at each port). This result emerges from the standard USEPA age-stratified exposure parameters: the children’s IR/BW ratio (1.0 L day^−1^/15 kg = 0.067) is 2.33-fold higher than the adult ratio (2.0/70 = 0.029), so even sub-threshold adult HI values translate to children’s HI above unity when the As concentration is moderately elevated. Adult HI values reported in isolation, which range from 0.50 to 0.67 in our dataset and fall well below the safety threshold, are therefore a less protective indicator than the age-stratified analysis. This pattern echoes the children-vs-adult exposure disparity reported for water-borne As by Miri et al. (2018) in northeast Iran, where children’s HI exceeded the safety threshold while adult HI did not; the present work, however, is (to our knowledge) the first Egyptian Red Sea port assessment to identify this exceedance, addressing a gap that Mohamedein et al. (2023) and earlier regional studies (El-Metwally et al., 2017) were not structured to detect.

Two qualifications limit the immediate operational interpretation of this result. First, oral ingestion of seawater may be a comparatively minor exposure pathway for the general population; the recreational scenario (incidental ingestion during swimming, ~ 50 mL per swimming event) yields per-event intakes considerably lower than the chronic 1 L day^−1^ assumption embedded in our calculation. The HI > 1 result should therefore be read as an upper-bound, screening-level signal under the most precautionary USEPA default rather than a direct prediction of clinical risk. Second, the present assessment is constrained to a single exposure route; integrated multi-pathway assessments (seafood ingestion, dermal uptake, inhalation of sea-spray aerosols) following Sayed et al. (2025) and Tavakoly Sany et al. (2024) would be needed to derive a population-relevant total daily intake. The seafood pathway is particularly important in this region: artisanal fisheries operate from Hurghada, Safaga, Nuweibaa and Sharm El-Sheikh, and inorganic As bioaccumulation in benthic invertebrates and demersal fish has been documented across the Red Sea (El-Sorogy et al., 2020).

A second source of conservatism in our health-risk numbers warrants explicit treatment. In 2025 the USEPA IRIS programme finalised a substantial reassessment of inorganic arsenic, revising the oral reference dose downward from 3.0 × 10^−4^ to 6.0 × 10^−5^ mg kg^−1^ day^−1^ (i.e. five-fold more stringent) and the oral cancer slope factor upward from 1.5 to 32 (mg kg^−1^ day^−1^)^−1^ (~ 21-fold more stringent). Applying the 2025 IRIS values as a sensitivity analysis, the adult HI_As_ component would rise from 0.481 to 0.651 to approximately 2.4–3.3, exceeding the threshold at every port; the children's HI would rise to approximately 5.7–7.6; and the As-driven carcinogenic risk would shift from 3.43 × 10^−5^ to 4.60 × 10^−5^ to approximately 7.3 × 10^−4^–9.8 × 10^−4^, crossing the 10^−4^ acceptable-maximum threshold at all six ports. We retain the legacy values in the primary computation to maintain direct comparability with the regional literature published before the IRIS revision (El-Sawy et al., 2023; El-Sorogy et al., 2024; Nour et al., 2022) while noting that future regional assessments (including any follow-up to this work) will need to adopt the 2025 values as a revised risk-assessment benchmark. The IRIS-2025 sensitivity test reinforces, rather than weakens, the precautionary conclusion drawn from the children’s HI exceedance.

Total carcinogenic risk under the legacy IRIS framework (3.43 × 10^−5^ to 4.60 × 10^−5^ for adults) is within the 10^−6^–10^−4^ acceptable range commonly cited in regulatory practice (Eke, 2025; USEPA United States Environmental Protection Agency, 1989), but it sits towards the upper end of this range. The As component drives the result; the Hg component contributes < 0.3%, reflecting both Hg’s modest oral slope factor and its low dissolved concentrations. This finding is consistent with the moderate, As-dominated CR profile reported for Hurghada coastal waters by El-Sorogy et al. (2024) and the broader Red Sea by Sayed et al. (2025).

### Limitations and recommendations for future work

Some limitations should be borne in mind when interpreting and extending the present results. The absence of Al, Fe or Sc as conservative lithogenic reference elements limits rigorous EF computation and hinders discrimination of lithogenic from anthropogenic inputs. Incorporation of these elements into future ICP-MS analyses, alongside grain-size and total organic carbon normalisation, would substantially strengthen source apportionment, particularly when combined with Positive Matrix Factorization (PMF) or APCS receptor modelling (Khaled-Khodja et al., 2024). The present health risk assessment addresses only the oral seawater-ingestion pathway; dermal contact during swimming, inhalation of marine aerosols at quay-side and seafood consumption (particularly relevant given the artisanal fisheries operating from Nuweibaa, Sharm El-Sheikh, Safaga and Hurghada) collectively contribute to total daily intake and should be integrated in subsequent assessments following the multi-pathway probabilistic framework of Sayed et al. (2025).

A targeted monitoring programme (biennial at minimum, ideally annual) covering all four elements addressed here plus speciated butyltins, with recommended inclusion of the children's exposure scenario and the IRIS-2025 toxicity values, is therefore a practical and operationally tractable recommendation for the Egyptian Environmental Affairs Agency and PERSGA (Regional Organization for the Conservation of the Environment of the Red Sea and Gulf of Aden).

## Conclusions

This study provides the first integrated, multi-index environmental and human health risk assessment for arsenic (As), selenium (Se), tin (Sn), and mercury (Hg) across six commercial and tourism ports of the Egyptian Red Sea coast. Environmental analyses revealed that seawater contamination is dominated by strong seasonal signals rather than spatial variation, with winter dissolved As averaging 3.2-fold higher than summer values due to reduced phytoplankton uptake and associated biogeochemical cycling, while Se exhibited the inverse pattern. In contrast, surficial sediments exhibited low contamination overall, with pollution load and ecological risk indices uniformly falling into low-risk categories. However, spatial hot spots were detected at Port-Tawfiq due to elevated As and Sn concentrations. Source apportionment identified two distinct anthropogenic clusters: a localized, petroleum-related Se signal at the Zayteiat oil terminal, and a persistent, co-occurring As–Sn–Hg signal associated with antifouling paints and remaining more than a decade after the 2008 IMO AFS Convention. The human health risk assessment presents a critical divergence between demographics. While adult hazard indices remained within safe limits, children’s hazard indices exceeded the safety threshold of 1.0 at every port, driven predominantly by As exposure. Sensitivity testing using updated 2025 USEPA IRIS toxicity values indicates that both non-carcinogenic and carcinogenic risks would exceed commonly used regulatory thresholds, underscoring the inadequacy of relying solely on legacy toxicity frameworks. As Suez Canal traffic recovers and intensifies contamination pressures, establishing an expanded, multi-pathway monitoring program is vital. Future initiatives must transition from the current baseline by integrating winter sediment sampling, conservative lithogenic reference elements (Al or Fe) for rigorous enrichment modeling, butyltin speciation, and multi-pathway probabilistic risk frameworks. These should encompass dermal, inhalation, and local seafood-consumption routes, calibrated to the revised 2025 IRIS toxicity values, to safeguard vulnerable coastal populations.

## Supplementary Information

Below is the link to the electronic supplementary material.

Supplementary file1 (DOCX 2848 KB)

## Data Availability

All raw seawater and sediment concentration data underlying this study are presented in Tables S1–S3 of the Supplementary Material. Derived contamination indices, USEPA risk calculations and the full Python analysis pipeline are available from the corresponding author on reasonable request.
