# Supplementary material for: Seasonal dynamics of trace elements, sediment contamination, and child health risks in six Egyptian red sea ports: an integrated water–sediment–human health assessment
Source: Environ Geochem Health. 2026 Aug 7;48(12):508. doi: 10.1007/s10653-026-03398-z (PMC13451294; doi:10.1007/s10653-026-03398-z)
Supplement: Supplementary file 1 [file 10653_2026_3398_MOESM1_ESM.docx]

**Supplementary Material**

**Seasonal Trace Element Dynamics, Sediment Contamination, and Children-Dominated Health Risk in Six Egyptian Red Sea Ports: An Integrated Water–Sediment–Human Assessment**

# Contents

Table S1. Station-level surface seawater concentrations — Summer 2017 (µg L^−1^).

Table S2. Station-level surface seawater concentrations — Winter 2018 (µg L^−1^).

Table S3. Station-level surficial sediment concentrations (µg g^−1^, dry weight).

Table S4. Complete contamination indices (CF, I_geo_, E_r_, RI) for all 19 stations.

Table S5. Paired t-test results for seasonal seawater comparisons.

Table S6. One-way ANOVA results comparing ports (annual means).

Table S7. Full Pearson correlation matrix for seasonal seawater variables.

Table S8. PCA loadings and explained variance for all eight principal components.

Fig. S1. Boxplots of seawater concentrations by port and season.

Fig. S2. Scatter plots of significant As–Sn, As–Hg and Sn–Hg co-variations in sediments.

Fig. S3. PCA scree plot and cumulative explained variance.

Fig. S4. Component loadings for PC1 and PC2.

Fig. S5. Geoaccumulation index profiles at all 19 stations.

Fig. S6. Decomposition of the non-carcinogenic hazard index by metal — adults vs. children.

Fig. S7. Station-level seawater concentration profiles (all 19 stations, both seasons).

**Table S1.** Station-level trace-element concentrations in surface seawater during Summer 2017 (µg L^−1^).

| **Station** | **Port** | **As** | **Se** | **Sn** | **Hg** |
| --- | --- | --- | --- | --- | --- |
| P1 | Port-Tawfiq | 3.834 | 0.402 | 0.474 | 0.115 |
| P2 | Port-Tawfiq | 2.601 | 0.357 | 0.431 | 0.115 |
| P3 | Port-Tawfiq | 3.991 | 0.446 | 0.647 | 0.096 |
| P4 | Port-Tawfiq | 2.242 | 0.223 | 0.345 | 0.135 |
| Z1 | Zayteiat | 3.587 | 0.580 | 0.474 | 0.096 |
| Z2 | Zayteiat | 2.197 | 0.536 | 0.474 | 0.115 |
| Z3 | Zayteiat | 2.332 | 0.402 | 0.517 | 0.115 |
| H1 | Hurghada | 2.646 | 0.379 | 0.603 | 0.154 |
| H2 | Hurghada | 2.466 | 0.455 | 0.560 | 0.192 |
| S1 | Safaga | 3.139 | 0.189 | 0.517 | 0.192 |
| S2 | Safaga | 2.265 | 0.227 | 0.388 | 0.231 |
| S3 | Safaga | 2.466 | 0.152 | 0.517 | 0.173 |
| S4 | Safaga | 2.556 | 0.114 | 0.474 | 0.192 |
| N1 | Nuweibaa | 2.399 | 0.268 | 0.603 | 0.115 |
| N2 | Nuweibaa | 2.960 | 0.268 | 0.474 | 0.173 |
| N3 | Nuweibaa | 2.489 | 0.402 | 0.733 | 0.192 |
| Sh1 | Sharm El-Sheikh | 2.578 | 0.134 | 0.560 | 0.212 |
| Sh2 | Sharm El-Sheikh | 2.825 | 0.313 | 0.690 | 0.269 |
| Sh3 | Sharm El-Sheikh | 2.848 | 0.223 | 0.991 | 0.192 |

**Table S2.** Station-level trace-element concentrations in surface seawater during Winter 2018 (µg L^−1^).

| **Station** | **Port** | **As** | **Se** | **Sn** | **Hg** |
| --- | --- | --- | --- | --- | --- |
| P1 | Port-Tawfiq | 9.931 | 0.156 | 2.692 | 0.167 |
| P2 | Port-Tawfiq | 9.783 | 0.208 | 2.564 | 0.417 |
| P3 | Port-Tawfiq | 9.980 | 0.182 | 1.538 | 0.292 |
| P4 | Port-Tawfiq | 12.451 | 0.208 | 1.923 | 0.208 |
| Z1 | Zayteiat | 9.783 | 0.286 | 3.846 | 0.125 |
| Z2 | Zayteiat | 9.347 | 0.182 | 2.436 | 0.292 |
| Z3 | Zayteiat | 9.654 | 0.156 | 1.538 | 0.250 |
| H1 | Hurghada | 7.461 | 0.156 | 2.179 | 0.167 |
| H2 | Hurghada | 7.762 | 0.130 | 1.410 | 0.250 |
| S1 | Safaga | 7.534 | 0.104 | 1.923 | 0.292 |
| S2 | Safaga | 8.232 | 0.156 | 4.103 | 0.250 |
| S3 | Safaga | 7.183 | 0.156 | 3.205 | 0.333 |
| S4 | Safaga | 7.461 | 0.182 | 1.410 | 0.583 |
| N1 | Nuweibaa | 9.931 | 0.130 | 1.026 | 0.083 |
| N2 | Nuweibaa | 9.041 | 0.234 | 1.154 | 0.182 |
| N3 | Nuweibaa | 8.255 | 0.182 | 1.667 | 0.083 |
| Sh1 | Sharm El-Sheikh | 8.624 | 0.156 | 1.923 | 0.125 |
| Sh2 | Sharm El-Sheikh | 8.135 | 0.182 | 2.949 | 0.182 |
| Sh3 | Sharm El-Sheikh | 8.057 | 0.208 | 1.410 | 0.208 |

**Table S3.** Station-level trace-element concentrations in surficial sediments (µg g^−1^, dry weight).

| **Station** | **Port** | **As** | **Se** | **Sn** | **Hg** |
| --- | --- | --- | --- | --- | --- |
| P1 | Port-Tawfiq | 14.61 | 0.161 | 8.633 | 0.158 |
| P2 | Port-Tawfiq | 7.40 | 0.182 | 1.265 | 0.034 |
| P3 | Port-Tawfiq | 17.50 | 0.121 | 6.857 | 0.198 |
| P4 | Port-Tawfiq | 7.49 | 0.182 | 0.851 | 0.039 |
| Z1 | Zayteiat | 7.33 | 0.121 | 1.633 | 0.039 |
| Z2 | Zayteiat | 7.30 | 0.252 | 0.879 | 0.048 |
| Z3 | Zayteiat | 7.66 | 0.211 | 1.286 | 0.058 |
| H1 | Hurghada | 2.20 | 0.161 | 0.398 | 0.015 |
| H2 | Hurghada | 3.68 | 0.153 | 1.269 | 0.018 |
| S1 | Safaga | 5.03 | 0.160 | 0.755 | 0.022 |
| S2 | Safaga | 7.60 | 0.201 | 0.382 | 0.019 |
| S3 | Safaga | 6.82 | 0.127 | 0.443 | 0.018 |
| S4 | Safaga | 5.27 | 0.151 | 0.578 | 0.015 |
| N1 | Nuweibaa | 5.34 | 0.214 | 0.428 | 0.018 |
| N2 | Nuweibaa | 5.11 | 0.070 | 0.282 | 0.015 |
| N3 | Nuweibaa | 1.79 | 0.232 | 0.512 | 0.018 |
| Sh1 | Sharm El-Sheikh | 5.22 | 0.171 | 0.450 | 0.015 |
| Sh2 | Sharm El-Sheikh | 3.35 | 0.111 | 0.717 | 0.018 |
| Sh3 | Sharm El-Sheikh | 9.23 | 0.214 | 0.604 | 0.020 |

# Statistical analyses

**Table S5.** Paired Student's t-test results comparing summer and winter dissolved trace-element concentrations across the 19 stations.

| **Element** | **Mean (S)** | **Mean (W)** | **t** | **df** | **p** |
| --- | --- | --- | --- | --- | --- |
| As | 2.734 | 8.819 | 20.08 | 18 | < 0.001 |
| Se | 0.304 | 0.176 | −4.86 | 18 | < 0.001 |
| Sn | 0.562 | 2.103 | 9.21 | 18 | < 0.001 |
| Hg | 0.163 | 0.224 | 2.04 | 18 | 0.056 |

**Table S6.** One-way ANOVA comparing annual mean trace-element concentrations among the six Egyptian Red Sea ports.

| **Element** | **F** | **df₁** | **df₂** | **p** | **Conclusion** |
| --- | --- | --- | --- | --- | --- |
| As | 0.26 | 5 | 33 | 0.93 | ns |
| Se | 2.48 | 5 | 33 | 0.053 | ns |
| Sn | 0.30 | 5 | 33 | 0.91 | ns |
| Hg | 1.98 | 5 | 33 | 0.11 | ns |

# Supplementary figures

**Fig. S1.** Boxplots of trace-element concentrations in surface seawater by port and season (n = 2–4 per port per season). Boxes show inter-quartile range; centre line is the median; whiskers extend to 1.5 × IQR; outliers shown as dots.

**Fig. S2.** Scatter plots of significant inter-element co-variations in surficial sediments (Pearson r; *** p < 0.001). Dashed lines: ordinary least-squares regression. Colour coding by port.

**Fig. S3.** Principal component analysis scree plot (a) and cumulative explained variance curve (b) for the eight seasonal trace-element variables in seawater (n = 19 stations).

**Fig. S4.** Component loadings for PC1 (a) and PC2 (b) from the PCA of seasonal seawater trace elements. Bars in green = positive loading; bars in red = negative loading.

**Fig. S5.** Geoaccumulation index (I_geo_) for As, Se, Sn and Hg at all 19 sampling stations across the six Egyptian Red Sea ports. Reference levels at I_geo_ = 1 (uncontaminated to moderately contaminated) and 2 (moderately to heavily contaminated) are shown as dashed lines.

**Fig. S6.** Decomposition of the non-carcinogenic hazard index (HI) by element, for adults (a) and children (b), via oral seawater ingestion. The dashed line indicates the threshold HI = 1. Note the elevated y-axis range in (b) and the HI > 1 outcome for children at every port.

**Fig. S7.** Station-level seawater concentration profiles across all 19 stations during Summer 2017 and Winter 2018 for (a) arsenic, (b) selenium, (c) tin and (d) mercury. Port groupings indicated by shaded background panels: PT = Port-Tawfiq, ZA = Zayteiat, HG = Hurghada, SF = Safaga, NW = Nuweibaa, SS = Sharm El-Sheikh.
